# Impact of Ser81 phosphorylation on alanine: glyoxylate aminotransferase associated with Primary hyperoxaluria type I

**DOI:** 10.1186/s43556-026-00442-3

**Published:** 2026-04-14

**Authors:** Sara Milosevic, Eduardo Salido, Noel Mesa-Torres, Angel L. Pey, Mario Cano-Muñoz

**Affiliations:** 1 Center for Rare Diseases (CIBERER), Hospital Universitario de Canarias, Universidad de La Laguna, San Cristóbal de La Laguna, Tenerife 38320 Spain; 2 Departamento de Química Física, Unidad de Excelencia en Química Aplicada a Biomedicina y Medioambiente E Instituto de Biotecnología, Universidad de Granada, Av. Fuentenueva S/N, Granada, 18071 Spain; 3 Department of Biotechnology and Environmental Protection, Estación Experimental del Zaidín, Consejo Superior de Investigaciones Científicas, Granada, 18008 Spain

**Keywords:** Alanine:glyoxylate aminotransferase (AGT), Primary hyperoxaluria type I (PH1), Phosphomimetic mutation, Protein localization, Post-translational modification

## Abstract

Phosphorylation is a fundamental post-translational modification that contributes to the dynamic control of protein function and stability. More than 300,000 site-specific phosphorylation sites have been detected across > 20,000 human proteins, yet only a small fraction (~ 5%) have been experimentally characterized, and their roles in health and disease remain largely unexplored. In particular, the functional consequences of most phosphorylation events in metabolic enzymes remain unknown. Here, we investigated the functional impact of modifying Ser81 in alanine:glyoxylate aminotransferase (AGT), the key enzyme responsible for glyoxylate detoxification and whose loss-of-function causes Primary Hyperoxaluria Type I (PH1). We examined phosphomimetic substitutions at Ser81 in the WT (wild-type) enzyme, the common polymorphic minor allele (LM, containing two variations, p.P11L and p.I340M), and the two most frequent PH1-associated variants (LM-p.G170R and LM-p.I244T). Using biochemical, biophysical and cell-based approaches, we found that introducing a negative charge at Ser81 (through the S81D substitution) strongly perturbs PLP (pyridoxal 5´-phosphate)/PMP (pyridoxamine 5´-phosphate) binding pose and disrupts catalytic activity, while preserving secondary and tertiary structure as well as peroxisomal localization. In contrast, the non-charged S81A substitution produced milder effects. These results indicate that Ser81 contributes to stabilizing the cofactor interaction network at the AGT active site. Our findings therefore identify Ser81 as a previously uncharacterized regulatory position that can critically influence AGT activity. Although further work is required to determine the physiological frequency and regulatory context of this modification in vivo, our results suggest that phosphorylation at this position could represent an additional modulatory layer influencing AGT function and genotype–phenotype relationships in PH1, with potential implications for understanding regulatory mechanisms affecting AGT activity in disease.

**Supplementary Information:**

The online version contains supplementary material available at 10.1186/s43556-026-00442-3.

## Introduction

Protein phosphorylation is one of the most widespread forms of post-translational regulation in human biology and contributes to the dynamic control of protein function, stability, interactions and localization [1–15]. These modifications likely have critical implications for both health and disease. Although hundreds of thousands of site-specific phosphorylation events have been detected through modern proteomics approaches, only a small fraction have been mechanistically characterized, and their functional relevance remains poorly understood. According to the PhosphoSitePlus® database [16] (accessed: 13 April 2025), approximately 0.5 million post-translational modifications (PTMs) have been reported across more than 20,000 human proteins using high-throughput methods. However, only about 5.3% of these PTMs have been characterized in detail. Notably, approximately 60% of all PTMs correspond to phosphorylation events on serine, threonine or tyrosine residues, and ∼36% specifically involve phosphorylation of serine residues. Despite this prevalence, the molecular consequences of most individual phosphorylation sites remain unknown. Understanding how specific phosphorylated residues influence protein structure, energetics and catalytic function is therefore essential for defining the regulatory complexity of human metabolic enzymes.

Alanine:glyoxylate aminotransferase (AGT) provides an informative system in which to investigate the mechanistic consequences of a defined phosphorylation event within a disease-relevant metabolic context. AGT is the key pyridoxal-5′-phosphate (PLP)-dependent enzyme responsible for detoxifying glyoxylate, and loss-of-function variants cause the rare metabolic disorder Primary Hyperoxaluria Type I (PH1) [17–19]. Numerous genetic variants of AGT have been characterized; however, the possible contribution of phosphorylation to AGT regulation has received very limited attention [15]. In the human population, the wild-type (WT) sequence accounts for approximately 80% of alleles, whereas about 20% contain the common minor allele (LM), defined by the polymorphisms p.P11L and p.I340M [20, 21]. Six potential phosphorylation sites have been reported for AGT, four of which correspond to tyrosine residues and cannot be readily investigated through conventional phosphomimetic mutagenesis strategies [22].

Among the reported phosphosites, Ser81 is of particular interest because it is positioned in close spatial proximity to the phosphate group of the PLP cofactor, and it has been detected in phosphoproteomics datasets curated in PhosphoSitePlus®. This location suggests that modification at this residue could directly influence cofactor binding and catalytic performance. However, the functional consequences of phosphorylation at this position have not been experimentally explored.

In this work, we used phosphomimetic mutagenesis as an experimental strategy to investigate the potential functional impact of Ser81 modification. Specifically, we introduced S81A and S81D substitutions and examined their effects across different AGT genetic backgrounds, including WT, the common polymorphic minor allele (LM), and two of the most frequent pathogenic variants associated with PH1 (LM-p.G170R and LM-p.I244T). Using biochemical, biophysical and cell-based approaches, we show that introducing a negative charge at position 81 (represented here by the phosphomimetic S81D substitution) strongly perturbs cofactor binding and catalytic activity while preserving overall protein structure and peroxisomal localization. These effects were consistent across all four genetic backgrounds tested, although the physiological balance between phosphorylated and non-phosphorylated AGT in vivo remains unknown. Together, our findings identify Ser81 as a previously unexplored regulatory position within the AGT active site and provide a mechanistic framework for understanding how post-translational modification at this site could influence AGT function and potentially contribute to genotype–phenotype relationships in PH1.

## Results

### p.S81D perturbs PLP binding and thermal stability while leaving the overall dimeric conformation largely unaltered

**Fig. 1:**
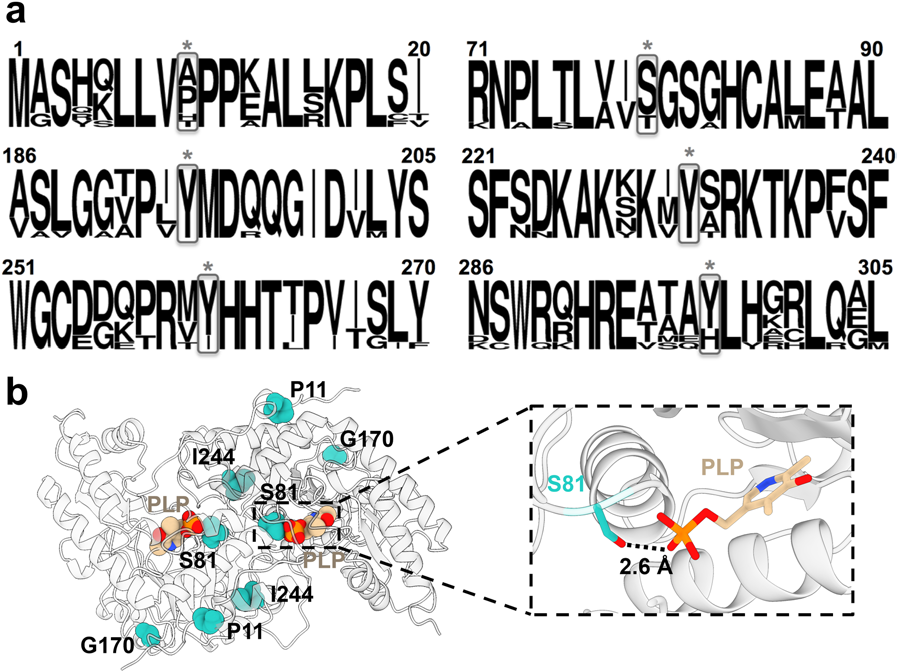
Selection of S81 as phosphorylation site to be studied in AGT variants associated with Primary hyperoxaluria type I. **a** Sequence-alignment statistical analyses of AGT sequences from mammals for the vicinity of the five phosphorylation sites described in Phosphosite Plus ® (from top to the bottom: T9, S81, Y194, Y231, Y260 and Y297). Sequences included were those from human, orangutan (*Pongo abelii*), monkey (*Macaca mulatta*), baboon (*Papio anubis*), dog (*Canis lupus familiaris*), horse (*Equus caballus*), cat (*Felis catus*), cow (*Bos taurus*), rabbit (*Oryctolagus cuniculus*), rat (*Rattus norvegicus*) and mouse (*Mus musculus*) enzymes. The figure was generated using WebLogo (https://weblogo.berkeley.edu/logo.cgi). **b** Structure of human AGT dimer (PDB 1H0C) highlighting the the location of P11, S81 (where the artificial phosphomimetic substitutions S81A and S81D were introduced), G170, I244 and the PLP molecule. The lower right panel shows a close-up view of the PLP-binding site, illustrating the short distance between S81 (2.6 Å between the side chain oxygen atom of S81 and one of the phosphate oxygens in PLP)

To evaluate whether the phosphomimetic modification at Ser81 affects the global structural integrity of AGT (Fig. 1) and its interaction with PLP across different genetic backgrounds an integrative biophysical characterization approach was used.

**Fig. 2:**
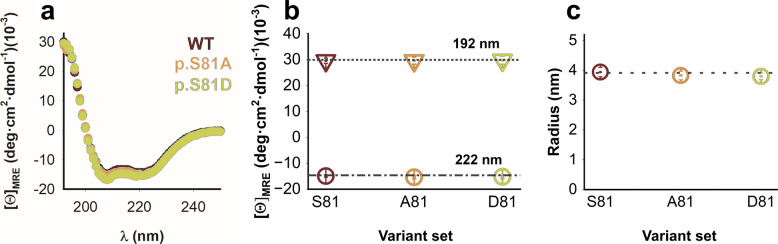
Overall conformation of AGT proteins. **a-b** Far-UV CD spectra for AGT proteins as purified (**a**). For each variant, spectra are the average from two different purifications. In panel **b**, we show data for the mean residue ellipticities at 192 nm and 222 nm (average ± s.d. for four AGT variants belonging to the S81, A81 and D81 sets, respectively). **c** Hydrodynamic radius for AGT proteins as purified from DLS analyses. Data are the average ± s.d. from n = 5 independent measurements. In **b** and **c**, the horizontal dotted lines indicate values for AGT WT as reference

Twelve AGT variants, AGT WT, LM, LM-p.G170R and LM-p.I244T (set S81) containing the mutation p.S81A (set A81) or p.S81D (set D81) were expressed in and purified from *E. coli* soluble extracts. These purified variants showed similar overall secondary structure (Fig. 2a-b and Fig. S1a) and similarly behaved as dimers in solution (Fig. 2c and Fig. S1b), indicating that the p.S81D and p.S81A mutations hardly affected the overall structure of the AGT dimer.

Binding of PLP to apo-AGT variants causes a dramatic increase in the thermal (conformational) stability of the protein, increasing the T_m_ by about 25 °C [23–26]. In addition, the LM variant showed lower stability than the WT protein, and this destabilization was enhanced by the p.G170R and p.I244T mutations primarily in the apo-state [23, 27]. Thermal denaturation profiles revealed striking differences between the S81/A81 and D81 sets (Fig. S2). In the apo-state, p.S81D consistently increased thermal stability across all four genetic backgrounds (average ΔT_m_ = + 7.7 ± 1.2 °C relative to S81 controls), whereas p.S81A showed only mild effects. (Fig. S2). In contrast, addition of excess PLP stabilized S81 but failed to stabilize D81 variants, which exhibited substantially reduced holo-state stability (ΔT_m_ = -23.1 ± 2.4 °C) (Fig. S2). The effects of the p.S81A mutation were much milder, with ΔT_m_ = -1.5 ± 0.5 °C and 2.4 ± 1.3 °C, for holo- and apo-AGT when these were compared with controls containing the variants p.S81 (Fig. S2). These data suggest that the p.S81D substitution stabilizes apo-AGT but interferes with the stabilizing interaction normally provided by PLP in all genetic backgrounds.

**Fig. 3:**
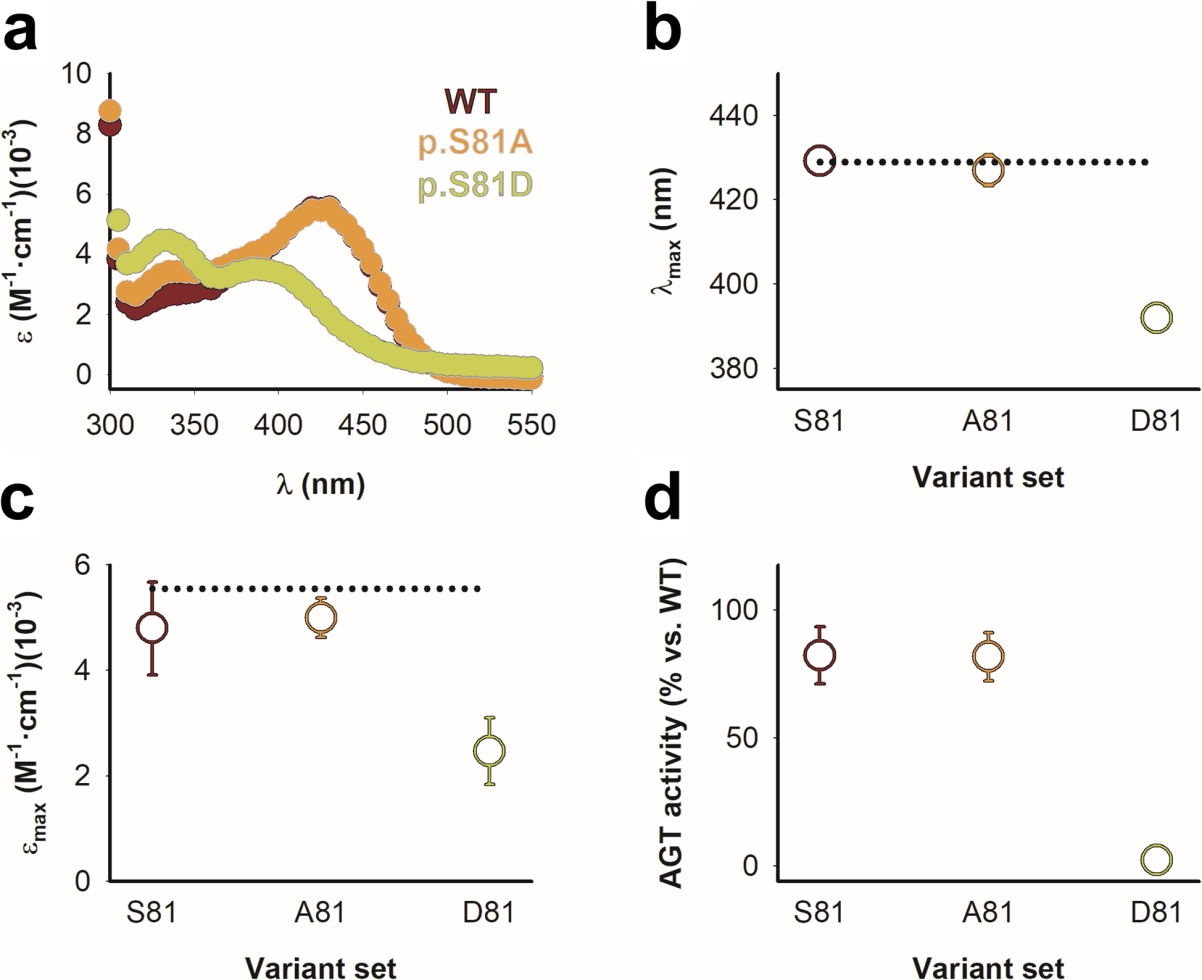
Spectroscopic analyses show that the p.S81D mutation distorts the PLP-binding conformation and abolishes AGT activity. **a** Near-UV/visible absorption spectra of AGT WT, p.S81A, and p.S81D variants. **b–c** Absorption maxima (**b**) and extinction coefficients at the maxima (**c**) for holo samples. **d** Overall transamination activity. Data represent mean ± s.d. from n = 5 for four genetic backgrounds (WT, LM, LM-p.G170R and LM-p.I244T) across the S81, A81, and D81 sets. Each activity value was normalized to WT AGT. Horizontal dashed lines in panels **b** and **c** indicate the corresponding values for WT AGT

Supporting this interpretation, spectroscopic analyses of the PLP bound to AGT variants provided additional evidence for functional alterations in the D81 set. Native AGT shows strong signatures of covalently bound PLP (forming a Schiff base with K209) including a characteristic absorption maximum at ~ 428 nm [23, 28, 29]. An unaffected binding pose of PLP can be identified from the spectral features in the absorption as well as its CD spectra (Fig. 3a-c and Fig. S3a-b). However, those variants containing p.S81D showed alterations in the electronic properties and local microenvironment of PLP. Variants in the S81 and A81 sets displayed maxima at 428 ± 3 nm (mean of eight variants). By contrast, all D81 variants exhibited pronounced blue shifts (mean ± s.d of ~ 392 ± 1 nm) and loss of the dichroic band associated with PLP, consistent with a perturbed electronic environment and distorted PLP-binding pose. In addition, CD spectra also suggested severely distorted binding of PLP to variants containing p.S81D, since the dichroic signal associated with bound PLP was absent for these mutants (Fig. S3b).

Together, these results show that the phosphomimetic substitution p.S81D maintains AGT global dimeric conformation intact but markedly disrupts PLP binding and PLP-mediated stabilization. The p.S81A substitution has milder effects, indicating that the major functional disruption arises from the introduction of a negative charge at position 81 rather than from simple removal of the Ser hydroxyl group. These findings support that phosphomimetic modification at Ser81 may impare cofactor engagement without inducing large structural changes.

### p.S81D abolishes AGT catalytic activity and severely impairs PLP → PMP conversion in the presence of L-alanine

To assess whether variants containing the phosphomimetic substitution p.S81D may affect the overall transaminase AGT activity, we carried out activity measurements with high concentrations of L-ala and glyoxylate as substrates and in the presence of an excess of PLP (Fig. 3d and S1c). Activity measurements revealed that all variants in the S81 and A81 sets showed comparable catalytic activity (1.39 ± 0.17 mmol Pyr·h^−1^·mg^−1^ protein). In contrast, activity in the D81 set was almost completely abolished (activity of 0.04 ± 0.05 mmol Pyr·h^−1^·mg^−1^ protein), consistent across the four genetic backgrounds tested. These results indicate that the introduction of a negatively charged residue at position 81 may be incompatible with normal catalytic turnover.

**Fig. 4:**
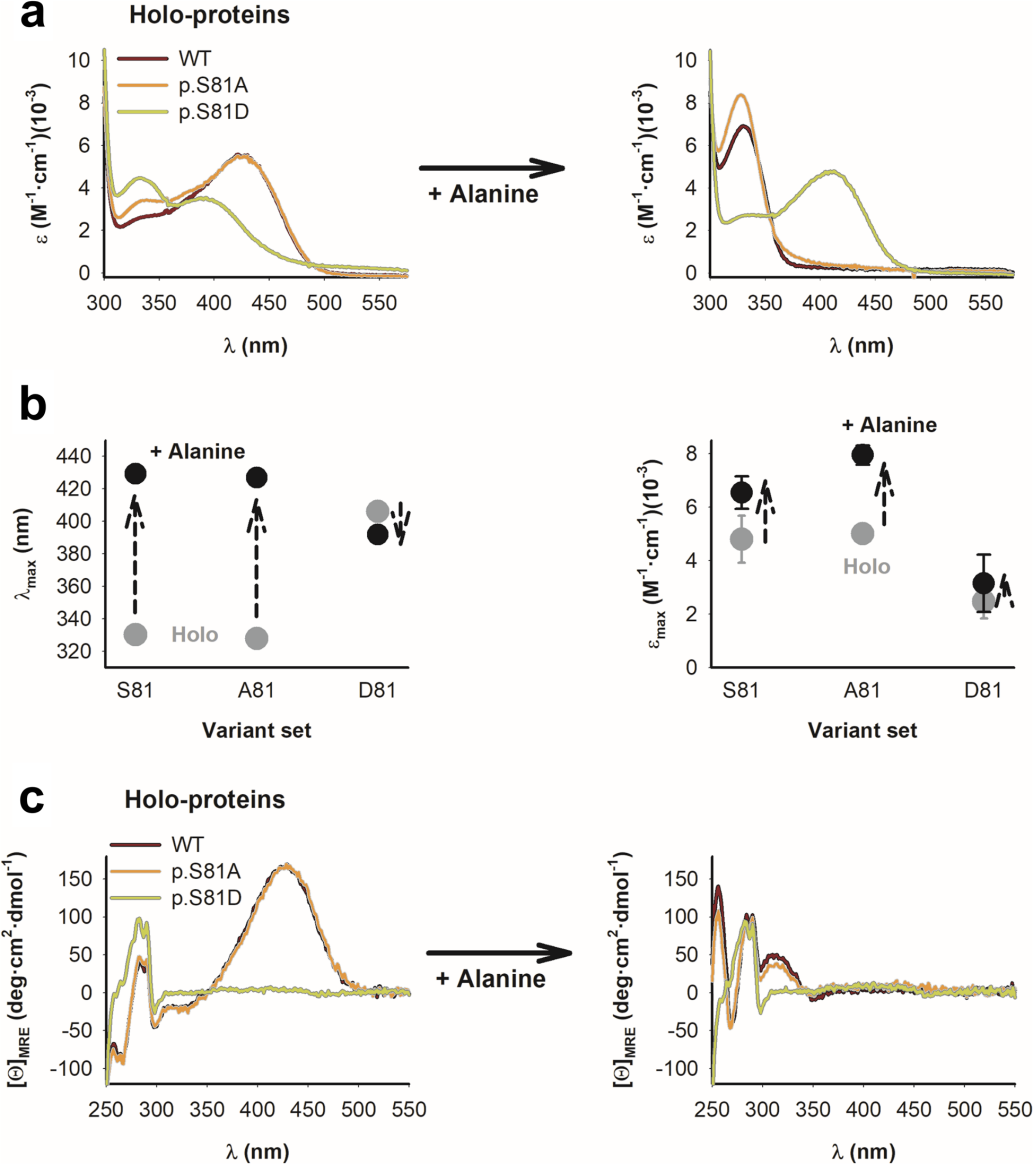
Spectroscopic analyses show that the mutation p.S81D prevents PLP-to-PMP transamination in the presence of L-Alanine. **a** Near-UV/Visible absorption spectra of AGT WT, p.S81A and p.S81D variants before (left panel) and after (right panel) addition of 200 mM L-Alanine. **b** Absorption maxima (left panel) and the extinction coefficient at the corresponding maxima (right panel) for holo samples before and after addition of 200 mM L-Alanine. Data are mean ± s.d. from n = 5 of four variants (WT, LM, LM-p.G170R and LM-p.I244T) across the S81, A81 and D81 sets. Vertical arrows indicate the spectral changes observed upon addition of L-alanine. **c** Near-UV/Visible CD spectra of AGT WT, p.S81A and p.S81D variants before (left panel) and after (right panel) addition of 200 mM L-Alanine. All spectra were corrected for dilution and represent averages of two independent experiments performed with two separate protein preparations for each variant

To test whether distorted PLP binding in D81 variants alters the first half-reaction of transamination (the conversion of PLP into Pyridoxamine 5´-phosphate, PMP) we analyzed changes in absorption and CD spectral signatures before and after incubation with an excess of L-alanine (Fig. 4a–c and Fig. S3a–b). In S81 and A81 variants, PLP was efficiently converted into PMP, as indicated by the disappearance of the characteristic PLP band at 428 ± 3 nm and the appearance of a strong absorption maximum at 329 ± 2 nm with increased extinction coefficient (from 4.9 ± 0.7 to 7.3 ± 0.9 mM^⁻1^·cm^⁻1^), consistent with previous characterizations associated with PMP formation (Fig. 4a-b and S3a) [28]. In contrast, D81 variants showed only minimal spectral changes upon incubation with L-alanine. Instead of the expected PLP → PMP transition, D81 variants displayed a modest red shift of the ∼392 nm band to 406 ± 2 nm, accompanied by a small and variable increase in extinction coefficient (from 2.5 ± 0.6 to 3.2 ± 1.1 mM^⁻1^·cm^⁻1^). Importantly, the CD signal associated with PMP formation, clearly present in S81 and A81 sets, was absent in all D81 variants (Fig. 4c and Fig. S3b). Thus, the absence of transaminase activity in these variants is most likely attributable to their impaired ability to catalyze amine transfer between the bound substrate and the PLP cofactor.

### p.S81D severely reduces PLP binding affinity, whereas p.S81A may reduce PMP binding affinity

We evaluated how phosphomimetic substitution at Ser81 influences the energetics and kinetics of cofactor binding. Because structural analyses indicated that p.S81D distorts the PLP-binding pose, we quantified how this substitution affects the association and dissociation of PLP and whether it influences the stability of the PMP-bound state. Conceptually, changes in PLP-binding energetics and kinetics may arise from effects on either the AGT–PLP complex or the unbound apo-enzyme. Perturbations to the bound complex would be expected to influence PLP dissociation rates (k_off_), whereas alterations in the unbound protein would primarily affect the association rate (k_on_). Although these interpretations are straightforward and consistent with our observations, PLP binding may involve additional intermediates or conformational states, and more complex scenarios cannot be excluded (see [30]).

**Fig. 5:**
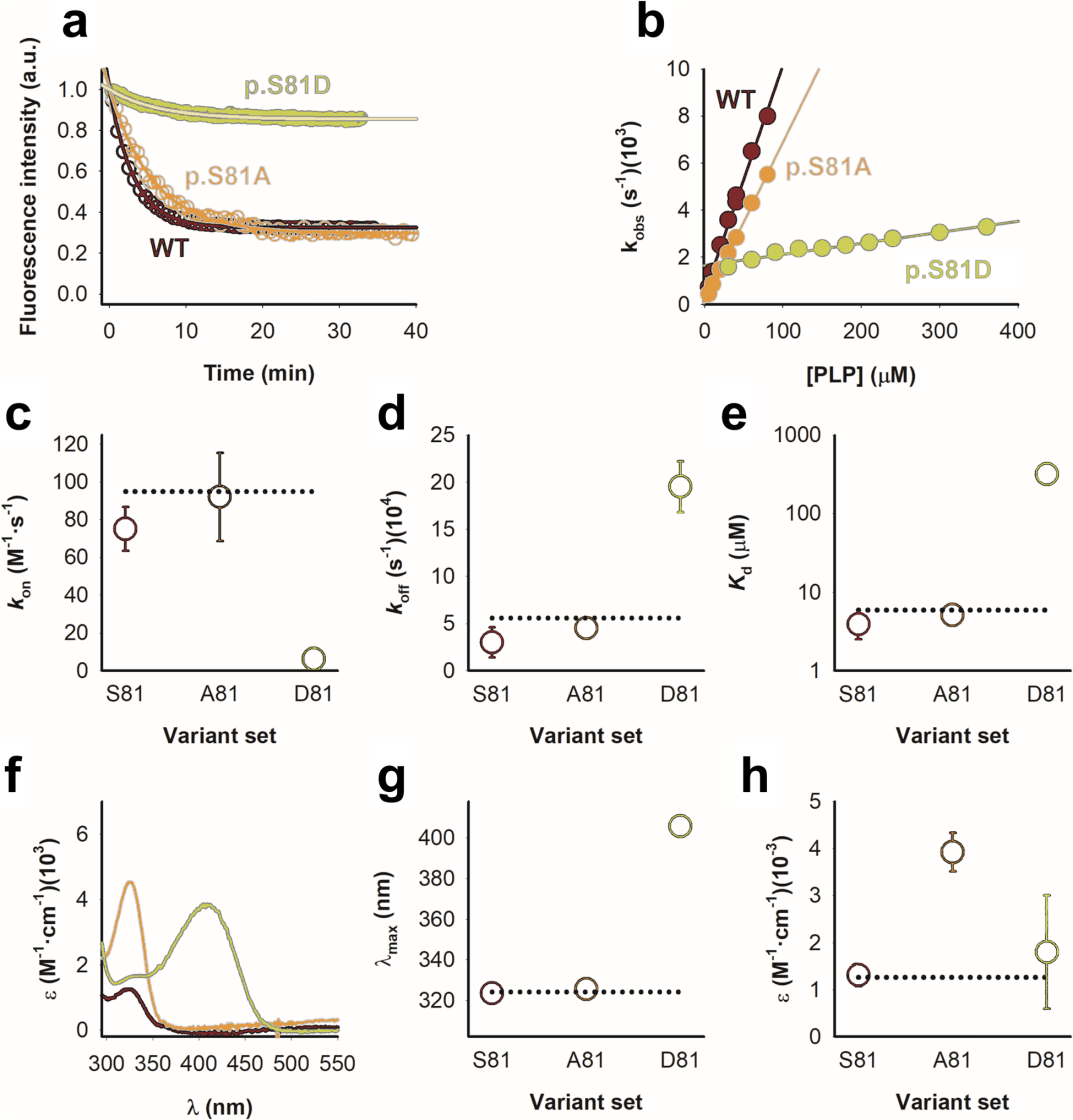
The mutation p.S81D markedly disrupts PLP binding affinity and kinetics whereas the mutation p.S81A primarily affects the stability of the PMP-bound state. **a** PLP binding kinetic traces (80 µM PLP and 0.4 µM apo-AGT) monitored by fluorescence spectroscopy (Exc. 280 nm, Em. 340 nm). Solid lines correspond tosingle-exponential fits used to obtain *k*_obs_. **b** Plots of *k*_obs_ vs. [PLP]. Linear fits were used to derive*k*_on_, *k*_off_ and *K*_d_ (Eqs. 1 and 2).**c-e** Values of *k*_on_ (**c**), *k*_off_ (**d**) and *K*_d_ (**e**) for AGT variants in the S81, A81 and D81 sets. Note the logarithmic scale in panel E. **f–h** Absorption spectra of filtrates obtained from reactions containing 20 µM holo-AGT and 200 mM L-Alanine. Panel **f** shows representative average spectra from two independent protein preparations for each variant. **g** and **h** show the wavelengths of the absorbance maxima (**g**) and the corresponding extinction coefficients (**h**). Data in panels **c**-**e** and **g**-**h** represent mean ± s.d. from n = 5 for four variants (WT, LM, LM-p.G170R, and LM-p.I244T) per set (S81, A81 and D81)

Consequently, we determined both rate constants as well as estimated the equilibrium dissociation constant (*K*_d_) for all variants from PLP binding kinetic experiments (Fig. 5a-b, Table S1). PLP binding showed markedly slower PLP association in D81 variants relative to S81 and A81 variants, with k_on_ values of 6.1 ± 1.2 for the D81 set vs 84 ± 21 M^−1^·s^−1^ for all S81/A81 variants (Fig. 5c). Correspondingly, dissociation rates were significantly faster in D81 variants (2.0 ± 0.3 ·10^–3^ s^−1^) compared to S81/A81 variants (3.7 ± 1.4 ·10^–4^ s^−1^)(Fig. 5d). Together, these effects resulted in a ~ 70-fold decrease in PLP binding affinity for D81 variants (K_d_ = 317 ± 71 μM) relative to S81/A81 variants (4.5 ± 1.2 μM; Fig. 5e), which implies a binding energy penalization of about 2.5 kcal·mol^−1^. These findings indicate that the negative charge introduced by p.S81D perturbs both the PLP-free state and the PLP-bound complex, simultaneously slowing binding (penalizing with ~ 1.5 kcal·mol^−1^) and accelerating dissociation of bound PLP (penalizing with about ~ 1 kcal·mol^−1^). We must note that the *K*_d_ value derived for AGT WT is approximately one order of magnitude lower than those determined by equilibrium titrations, possibly due to higher uncertainty in the determination of *k*_off_ (as previously shown [28]). Nevertheless, our data are consistent with p.S81D mutation markedly slowing down PLP binding association and accelerating dissociation, resulting in a substantially reduced binding affinity.

Additional spectroscopic analyses following the first half of the transamination cycle showed that PLP bound to variants containing p.S81D was readily released from AGT-PLP complex (Fig. 5f-g and Fig. S3c). Interestingly, variants containing p.S81A exhibited altered PMP release compared with native Ser81 (Fig. 5h). While the wavelength of the released PMP maximum after incubation of AGT variants with L-alanine is not significantly affected by p.S81A (324 ± 1 for S81 vs 326 ± 1 nm for A81 variants), the amount of PMP released from these complexes seems threefold higher for the variants containing p.S81A. Considering a ε_325_ for PMP of 8300 M^−1^·cm^−1^ [31], a fraction of PMP released per AGT monomer of 0.47 ± 0.06 is calculated for p.S81A variants, compared to 0.16 ± 0.03 for S81 variants.

These kinetic and spectroscopic data demonstrate that the phosphomimetic substitution p.S81D severely compromises PLP binding by both slowing association and accelerating dissociation, leading to greatly reduced affinity. In contrast, p.S81A has minimal impact on PLP binding but modestly alters PMP release after catalysis.

### Structural modeling of p.S81A and p.S81D variants supports their effects on PLP and PMP binding and catalysis

**Fig. 6:**
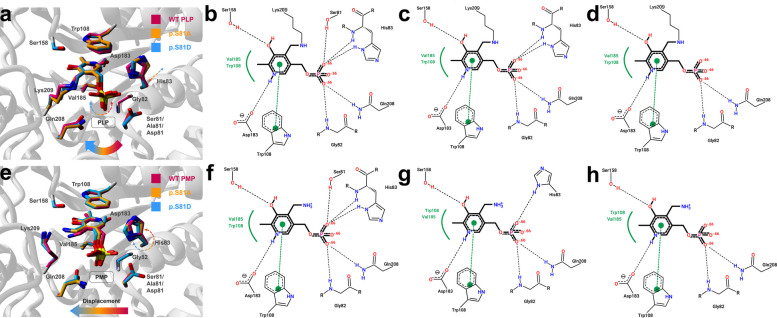
Structural modeling of AGT interactions with PLP (panels a-d) and PMP (panels e–h). **a **Close-up view of the AGT–PLP complex. PLP and interacting side chains are shown as sticks, with the color code indicating WT (magenta), p.S81A (orange), and p.S81D (blue) models. Panels **b**–**d** show 2D interaction diagrams of PLP and its neighboring residues in the active site (**b** WT; **c** p.S81A; **d** p.S81D). **e** Close-up view of the AGT-PMP complex, displayed as in panels **a**–**d**. **f**–**h** show 2D interaction diagrams of PMP and surrounding residues (**f** WT; **g** p.S81A; **h** p.S81D). In panels **a** and **e**, thin arrows highlight structural perturbations induced by the substitutions. In the 2D diagrams, hydrogen bonds are shown as black dashed lines, and hydrophobic contacts are depicted by residue labels with spline segments along the interacting hydrophobic surfaces

To characterize how the p.S81A and p.S81D substitutions affect PLP and PMP binding and their consequences for catalysis, we analyzed the AGT–PLP crystal structure (PDB 1H0C) and generated corresponding AGT–PMP models incorporating each substitution.In the AGT–PLP complex (Fig. 6a–d), PLP is covalently linked to K209, restricting its mobility within the active site. Modeling of p.S81A showed only minor perturbations relative to the WT structure. PLP exhibited a small pivoting movement toward W108, accommodated by a coupled adjustment of the H83 side chain, which preserved interactions between H83 and the PLP ring system (Fig. 6c). In contrast, p.S81D introduced a negatively charged side chain adjacent to the PLP phosphate group, generating electrostatic repulsion that displaced PLP further from residue H83 (Fig. 6d). This shift prevented formation of the hydrogen bond between H83 and PLP observed in WT and p.S81A models and reduced the number of stabilizing contacts within the active site.

In the AGT-PMP complex, p.S81 forms a hydrogen bond with phosphate group from PMP, as observed for the PLP in the crystal structure (Figs. 1b, 6b and f). This interaction may hold PMP in place within the enzyme active site and positions it in an optimal conformation. This interaction was absent in the p.S81A model, allowing PMP greater mobility. Loss of the Ser81–phosphate hydrogen bond weakened the adjacent interaction between PMP and H83, resulting in a ~ 21° reorientation of PMP relative to its WT pose (Fig. 6e and g). The effects were more pronounced in the p.S81D model. Introduction of the negative charge at position 81 displaced PMP even further from H83, disrupting their hydrogen bond entirely and leading to a larger reorientation (~ 37° rotation) of the cofactor (Fig. 6e and h). This shift decreased the number of hydrogen bonds stabilizing the PMP-bound state. Interestingly, the altered electrostatics repositioned the H83 side chain by ~ 14° (around its C_α_-C_β_ bond), allowing it to form a compensatory hydrogen bond with Asp81, which may help stabilize the N-terminal region of helix α5.

### AGT colocalizes with the peroxisomal marker PMP70 in both the S81 and D81 variants.

AGT primarily localizes to peroxisomes, where it performs its metabolic function [20, 21, 32–34]. Proper peroxisomal targeting is essential for AGT function, and mislocalization to mitochondria is a well-documented pathogenic mechanism in PH1, particularly in certain disease-associated variants such as p.G170R and p.F152I, which are known to cause mistargeting [35]. Given that phosphorylation can influence protein trafficking in other systems, we sought to determine whether the phosphomimetic substitution at Ser81 affects the subcellular localization of AGT.

**Fig. 7:**
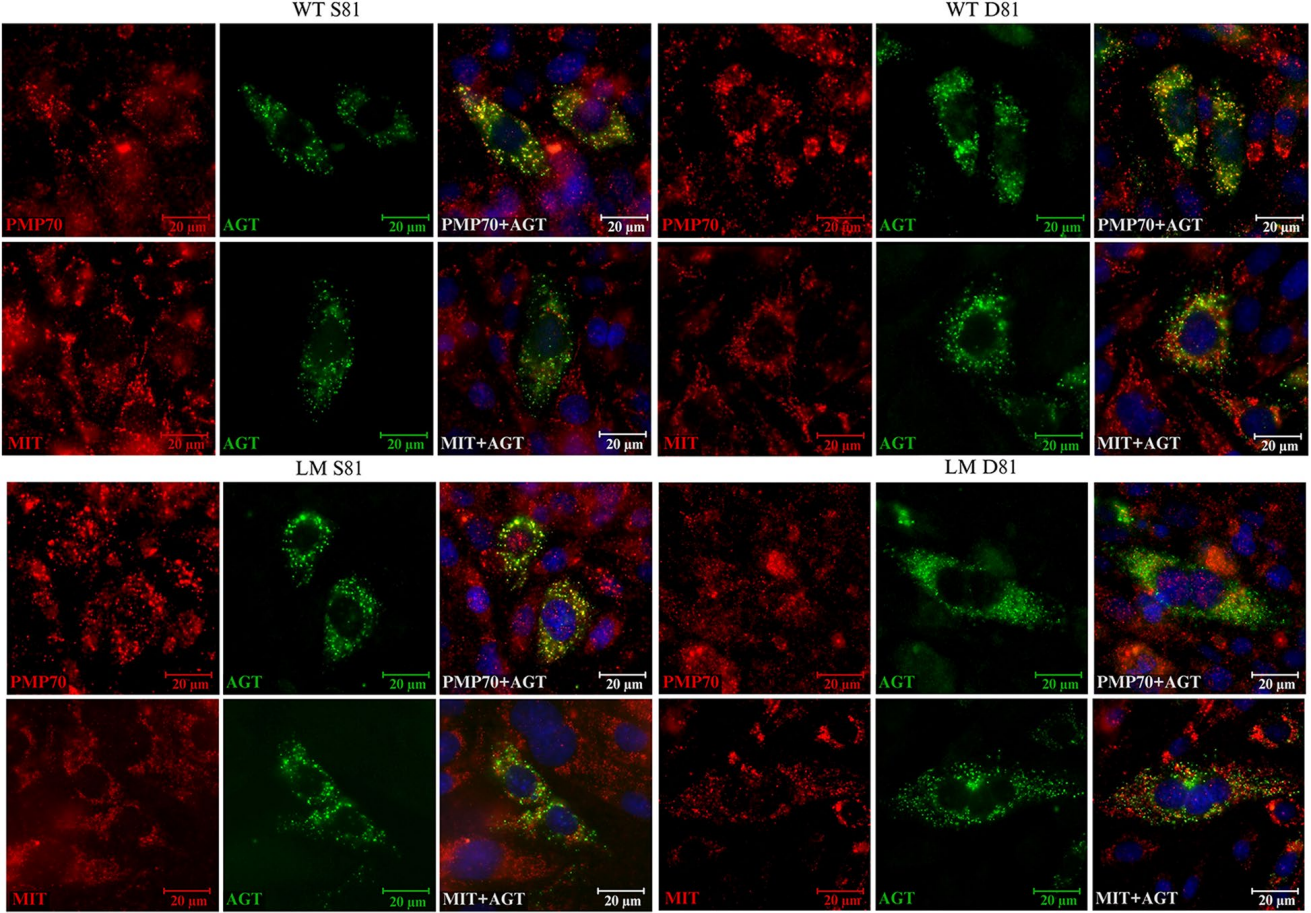
The phosphomimetic mutation does not affect AGT peroxisomal localization. AGT is shown in green while peroxisomes or mitochondria are labeled in red. Colocalization with the peroxisomal marker PMP70 appears as a yellow signal in merged images, indicating proper targeting to peroxisomes. All four variants displayed strong peroxisomal colocalization, with no evidence of mitochondrial mistargeting. The intracellular distribution of AGT was comparable between WT and LM samples

To test whether the p.S81D mutation affects the intracellular location of AGT, we carried out immunofluorescence colocation studies in CHO cells transfected with WT and LM AGT constructs, with or without the p.S81D substitution. AGT was visualized using a specific anti-AGT antibody (green), while peroxisomes and mitochondria were labeled with anti-PMP70 and mitochondrial antibody, respectively (red) (Fig. 7). To complement the imaging data, we also quantified the extent of AGT–PMP70 colocalization through quantitative colocalization analysis (Table S2). In all conditions tested, AGT colocalized extensively with the peroxisomal marker PMP70, producing a characteristic yellow signal in merged images (Fig. 7). No AGT signal significantly overlapped with mitochondrial staining, and the pattern was consistent between WT and LM backgrounds. AGT was predominantly localized in peroxisomes, with no significantly detectable mitochondrial localization. Quantitative colocalization analysis further supported these observations. Pearson’s correlation coefficients and Mander’s overlap coefficients indicated high AGT–PMP70 colocalization for all variants, with no statistically significant differences between S81 and D81 constructs.

Immunofluorescence imaging and quantitative colocalization analyses indicate that the phosphomimetic substitution p.S81D does not disrupt AGT peroxisomal targeting in either the WT or LM background. Within the limitations of this assay, these data suggest that modification at Ser81 affects catalytic function without altering subcellular localization. Because immunofluorescence provides only limited resolution, these observations should be interpreted cautiously, and more detailed localization approaches, such as subcellular fractionation or high-resolution imaging techniques such as immunoelectron microscopy, will be required in future studies to fully validate the absence of mistargeting.

## Discussion

In this study, we investigated how substitutions at Ser81 influence the biochemical properties of AGT across four genetic backgrounds (WT, LM, LM-p.G170R and LM-p.I244T). Ser81 has been identified as a phosphorylation site in large-scale human phosphoproteomics datasets curated in PhosphoSitePlus®, yet its functional relevance has not been characterized. Because the phosphorylated form of AGT was not experimentally accessible, we used phosphomimetic substitutions, p.S81A and p.S81D, to approximate the biochemical consequences of hydroxyl removal or introduction of a negative charge at this position.

Our structural, kinetic, and spectroscopic analyses consistently indicate that introducing a negative charge at position 81 (p.S81D) perturbs PLP and PMP binding without altering the global fold or dimeric assembly of AGT. Thermal denaturation experiments showed that p.S81D stabilizes apo-AGT but prevents the strong PLP-mediated stabilization normally observed in WT and LM variants. Kinetic measurements revealed that p.S81D decreases PLP affinity by ∼70-fold by slowing association and accelerating dissociation, consistent with the distorted PLP-binding pose detected by UV–visible and CD spectroscopy. These biochemical findings were supported by structural modeling, which predicted that p.S81D displaces PLP and PMP from key stabilizing residues, particularly H83, and disrupts the hydrogen-bonding network surrounding the cofactor phosphate. Together, these data support a model in which a negatively charged substitution at Ser81 destabilizes both the PLP-bound and PMP-bound conformations, thereby impairing the first half-reaction of transamination and nearly abolishing catalytic activity. In contrast, p.S81A produced relatively mild effects. An important observation is that these biochemical effects were consistent across all four genetic backgrounds, including both non-pathogenic (WT, LM) and disease-associated variants (LM-p.G170R, LM-p.I244T). Because AGT mistargeting to mitochondria is a defined pathogenic mechanism in PH1, we evaluated whether p.S81D affects subcellular localization. Immunofluorescence imaging and quantitative colocalization analyses demonstrated that AGT remained localized at peroxisomes in both S81 and D81 constructs. While our data indicate no significant mistargeting, more detailed localization studies will be required to validate this conclusion.

Future work will be needed to establish the physiological and clinical relevance of our findings. An important next step will be to determine whether Ser81 is phosphorylated in vivo under specific metabolic or regulatory conditions, and to characterize its abundance in healthy and PH1 contexts using targeted phosphoproteomics. Additional studies should also examine how modification at this residue influences downstream metabolic outcomes, including glycolate and oxalate handling, in cellular or animal models that more closely reflect the physiological environment. Finally, extending the analysis to a broader panel of vitamin B6 vitamers (e.g., pyridoxine, PN, pyridoxal, PL, pyridoxine 5'-phosphate, PNP) will be of relevance to fully define how perturbations at Ser81 affect cofactor specificity and binding energetics across the entire B6 metabolic landscape.

Despite these limitations, our findings suggest that post-translational modification at Ser81 could represent an additional layer of AGT regulation, potentially influencing cofactor engagement independently of folding and peroxisomal targeting showing functional, structural and electrostatic changes that resemble well those described for 5´-phosphate in p.G82E [28] and LM-p.H83R [23]. Notably, two natural missense variants at this position (p.S81W and p.S81L) have been reported in ClinVar, including one observed in a PH1 patient, although no functional studies are available. Our work provides a mechanistic rationale for why post-translational modifications at this site may be deleterious. If validated in future cellular and in vivo studies, the disruption of cofactor binding caused by modification at Ser81 may complement existing models of genotype–phenotype relationships in PH1, which currently focus on protein stability, folding, and mistargeting. This marked impairment of cofactor engagement caused by a phosphomimetic substitution at Ser81 suggests that post-translational modifications at this residue could influence AGT function in vivo in ways not captured by current diagnostic workflows. If future studies confirm that dysregulated phosphorylation at Ser81 (or at other AGT phosphosites) occurs in patients compared to healthy population, such modifications might contribute to functional AGT deficiency even in the absence of coding mutations, with potential value as biomarkers of enzymatic competence. In this scenario, monitoring AGT phosphorylation status or targeting the responsible kinase/phosphatase pathways could eventually complement existing diagnostic and therapeutic strategies for PH1.

## Methods and materials

### Protein expression and purification

To study the effects of phosphorylation point mutations converting the phosphorylatable Ser81 to either alanine (as control) or aspartate (negatively charged amino acid) were generated in the pCOLDII constructs encoding AGT WT, LM, LM-p.G170R or LM-p.I244T [23] using a site-directed mutagenesis approach. The presence of the desired substitutions and the integrity of the entire coding sequence were verified by Sanger sequencing. The resulting plasmids were transformed into *E. coli* BL21(DE3) cells for recombinant protein expression. Cultures were induced with 0.4 mM IPTG and maintained at 4 °C for approximately 4–6 h to promote soluble expression. Cell lysates were clarified and the AGT variants were isolated by nickel-based affinity purification, followed by size-exclusion chromatography, following procedures adapted from refs. [23, 24]. Apo-forms of the enzymes were obtained by incubation with L-alanine under mildly acidic conditions, as described previously [24]. Protein preparations were stored in 20 mM Na-HEPES buffer containing 200 mM NaCl at pH 7.4. Concentrations were determined from absorbance at 280 nm using a monomer extinction coefficient of 47,000 M^⁻1^·cm^⁻1^.

### Overall transamination activity measurements

Transaminase activity was assessed by monitoring pyruvate generation under defined conditions. Purified AGT samples were used at concentrations typically ranging between 2.5–4 µg·ml^⁻1^, although assays involving the p.S81D variants required up to ~ 50 µg·ml^⁻1^ due to their low catalytic output. Reactions were conducted in potassium phosphate buffer (100 mM, pH 8.0) containing glyoxylate (10 mM), L-alanine (100 mM) and an excess of PLP (150 µM). Mixtures were incubated at 37 °C for 2 min and then immediately brought to a stop by adding trichloroacetic acid to a final concentration of 25% (w/v). Samples were clarified by centrifugation (approximately 21,000 × g, 10 min, 4 °C), and the supernatants were kept frozen until analysis.

Pyruvate levels in the deproteinized samples were quantified enzymatically using lactate dehydrogenase in the presence of 0.2 mM NADH at 37 °C. The consumption of NADH was followed spectrophotometrically at 340 nm in 1 M Tris–HCl (pH 8.0) using quartz cuvettes in an Agilent 8453 spectrophotometer operated at 25 °C. Concentrations were obtained by comparison with calibration curves generated from pyruvate standards prepared and measured under identical assay conditions. Each AGT construct was analyzed in at least four independent experiments, and activity values are reported as mean ± standard deviation.

### Spectroscopic and light scattering measurements

Unless otherwise specified, all optical measurements were performed in a buffer consisting of 20 mM HEPES (sodium salt), 200 mM NaCl, pH 7.4. UV–visible absorption spectra were recorded at 25 °C using an Agilent 8453 diode-array spectrophotometer, with samples placed in standard 1-cm quartz cuvettes. AGT variants were analyzed at a concentration of 20 µM. To monitor formation of PMP, L-alanine was added to a final concentration of 0.2 M, and samples were incubated for at least 10 min at 25 °C prior to data acquisition. To evaluate whether PMP dissociated from the enzyme after this reaction, mixtures were passed through 30-kDa centrifugal filter units (VIVASPIN® 6, Sartorius), and the absorbance spectra of the resulting filtrates were collected under the same conditions.

Circular dichroism (CD) experiments were conducted on a Jasco J-710 instrument equipped with Peltier temperature control, operating at 25 °C. Samples for near-UV/visible CD were prepared as described for the absorption assays. Spectra were collected at 100 nm·min^⁻1^ using a 2-nm bandwidth, and five consecutive scans were averaged; measurements were carried out in 5-mm path-length quartz cuvettes, and corresponding buffer blanks were acquired and subtracted. Far-UV CD spectra were obtained using AGT samples diluted to 5 µM in 20 mM potassium phosphate (pH 7.4). These spectra were acquired at the same scanning speed with a 1-nm bandwidth, using 1-mm cuvettes; six scans were averaged for each variant. In all cases, two independent protein preparations were analyzed and averaged for the final datasets.

Dynamic light scattering (DLS) was performed on a DynaPro MSX instrument (Wyatt Technology). Measurements were carried out at 25 °C in a micro-volume cuvette (1.5 mm path length) using 10 µM AGT samples. For each analysis, 25 acquisitions were collected and averaged, and hydrodynamic radii were estimated assuming spherical particles using the Stokes–Einstein equation. Reported values represent the mean ± standard deviation from 4–6 independent measurements.

PLP-binding kinetics were determined for apo-proteins under pseudo–first-order conditions, using PLP concentrations in the range of 5–100 µM, always in large excess relative to apo-AGT (typically 0.4 µM / monomer). Prior to initiating reactions, samples were equilibrated at 25 °C for 5 min in 1-cm quartz cuvettes. PLP was then added to the desired concentration and mixed manually; the dead time of manual mixing (10–40 s) was recorded and incorporated into the kinetic analysis. Time-dependent fluorescence emission was monitored on a Cary Eclipse fluorimeter (Varian) using excitation at 280 nm and emission at 340 nm (5-nm bandwidths). The resulting binding traces were fitted to a single-exponential equation to obtain the observed rate constant (k_obs_). Under pseudo–first-order conditions, k_obs_ reflects the association equilibrium constant (K_a_), the second-order association rate (k_on_) and the first-order dissociation rate (k_off_) according to standard kinetic relationships:

1 $${k}_{obs}={k}_{off}+ {k}_{on}\cdot [PLP]$$

2 $${K}_{a}=\frac{{k}_{on}}{{k}_{off}}$$

where [PLP] is the total concentration of cofactor. Plotting the observed rate constants against the PLP concentration and fitting the data to the relationships described above allowed us to extract the apparent kinetic and equilibrium parameters governing PLP association and dissociation, together with their fitting uncertainties. The dissociation constant (K_d_) was obtained as the reciprocal of the association equilibrium constant (K_d_ = 1/K_a_).

### Thermal stability

Thermal unfolding profiles of AGT variants were obtained by monitoring intrinsic fluorescence changes during controlled heating. Protein samples (1 µM, expressed as monomer concentration) were prepared either in the holo form, by supplementing purified enzyme with 100 µM PLP, or as apo proteins generated as described in earlier sections. All measurements were performed in a buffer composed of 20 mM HEPES and 200 mM NaCl at pH 7.4, using 1 cm quartz cuvettes.

After equilibrating each sample at 20 °C for approximately five minutes, temperature ramps were applied from the starting temperature up to 70–95 °C, depending on the stability of the variant, at a heating rate of 2 °C per minute. Fluorescence emission was recorded on a Varian Cary Eclipse spectrofluorimeter using excitation at 280 nm and monitoring emission at 360 nm (bandwidths set to 5 nm).

Raw fluorescence traces were corrected by subtracting linear baselines obtained from pre- and post-transition regions. The apparent melting temperature (T_m_) was defined as the midpoint of the unfolding transition. For each construct, three to four independent measurements were carried out, and the resulting T_m_ values are reported as mean ± standard deviation.

### Molecular modeling

Because no experimental structure is available for the PMP-bound form of human AGT, we generated a model of this state. Starting from the crystal structure of holo-AGT (PDB 1H0C) [29], PMP was manually positioned in the active site by reproducing the orientation and interactions observed for PLP, while retaining the crystallographic water network. The initial complex was subjected to an energy-minimization step, following procedures adopted in earlier studies [25, 28]. During this stage, all non-hydrogen atoms were kept fixed so that the added hydrogens could adjust to the local environment. Subsequently, the PMP molecule and side chains of residues located within a 20 Å radius of the cofactor were allowed to relax.

Single amino-acid substitutions at position 81 (S81A and S81D) were then introduced using YASARA modeling suite [36, 37]. After introducing each mutation, the structures were refined to remove steric clashes and to correct covalent geometry by energy minimization with the NOVA force field [38]. A cutoff of 8 Å was employed for nonbonded interactions, and long-range electrostatics were modeled using the Particle Mesh Ewald method [39, 40]. Following an initial steepest-descent minimization to eliminate high-energy contacts, the system underwent simulated annealing (2 fs integration steps; velocity scaling by a factor of 0.9 every ten steps) until the energy stabilized, defined as an improvement of < 0.05 kJ·mol⁻^1^ per atom over 200 consecutive steps.

Schematic two-dimensional interaction diagrams highlighting the contacts between PMP and surrounding residues were produced using PoseView [41]. Hydrogen bonds are depicted as dashed lines, and nonpolar interactions are shown through annotated residues and spline segments following the hydrophobic regions of the ligand.

### Experiments in CHO cells

Chinese hamster ovary (CHO) cells (ATTC, USA) were grown in alpha-minimal essential medium (α-MEM, Lonza, Germany) supplemented with glutamine, penicillin/streptomycin and 5% fetal bovine serum, on 13 mm glass coverslips placed in 6-well plates (Avantor, Spain). Cell transfections were performed using AGT cDNA variants subcloned in pCIneo plasmids (Promega, USA) with FectoCHO reagent (Sartorius, Germany), following manufacturer’s guidelines.

The following AGT variants were tested: S81 in both major (WT) and minor (LM) alleles, and D81 in both major (WT) and minor (LM) alleles. After 24 h, cells were fixed with buffered formaline (Avantor, Spain) for 10 min, permeabilized with 0.1 triton-X and immunofluorescence was performed as previously described [23]. Cells were labeled with two different sets of antibodies: a) guinea pig anti-AGT (1:1000) and rabbit anti-PMP70 (1:1000) to assess colocalization of AGT and the peroxisomal marker; and b) guinea pig anti-AGT (1:1000) and rabbit anti-mitochondria (1:5000) to assess colocalization of AGT and the mitochondria. After 1 h incubation with primary antibodies, coverslips were washed with phosphate buffered saline (PBS) and incubated for 30 min. With the secondary antibody mix (Alexa Fluor-488 goat anti-guinea pig IgG and Alexa Fluor-555 goat anti-rabbit IgG). After PBS wash, coverslips were mounted on glass slides with PBS-glycerol containing Hoesch-33342 to counterstain the nuclei. Digital images were acquired with a fluorescence microscope (Zeiss, Germany) using a 60 × objective with immersion oil and sequential blue-green-red channel emission filters. Image analysis was performed with Fiji version of ImageJ software (NIH) using Coloc 2 plugin for colocalization analysis.

## Supplementary Information

Supplementary Material 1.

## Data Availability

The authors declare that the data supporting the findings of this study are available within the paper and its Supplementary Information files. Should any raw data files be needed in another format they are available from the corresponding author upon reasonable request.

## Abbreviations

AGT: Alanine:glyoxylate aminotransferase 1
CD: Circular dichroism
CHO: Chinese ovary hamster
DLS: Dynamic Light Scattering
k_obs_: Observed rate constant
HEPES: 4-Hydroxyethylpiperazine ethanesulfonic acid
K_a_: Apparent equilibrium association constant
K_d_: Apparent equilibrium association constant
k_off_: Apparent dissociation rate constant
k_on_: Apparent association rate constant
PBS: Phosphate buffered saline
PH1: Primary hyperoxaluria type I
PMP: Pyridoxamine 5´-phosphate
PLP: Pyridoxal 5´-phosphate
PN: Pyridoxine
PL: Pyridoxal
PNP: Pyridoxine 5'-phosphate
PTM: Post-translational modification
*T*_m_: Apparent denaturation temperature
WT: Wild type
LM: Minor allele of alanine:glyoxylate aminotransferase 1
