# Supplementary material for: Impact of Ser81 phosphorylation on alanine: glyoxylate aminotransferase associated with Primary hyperoxaluria type I
Source: Mol Biomed. 2026 Apr 14;7:51. doi: 10.1186/s43556-026-00442-3 (PMC13079258; doi:10.1186/s43556-026-00442-3)
Supplement: Supplementary file 1 — Supplementary Material 1. [file 43556_2026_442_MOESM1_ESM.docx]

**Supplementary Information for article:**

**Impact of Ser81 Phosphorylation on alanine:glyoxylate aminotransferase Associated with Primary Hyperoxaluria Type I**

Sara Milosevic^1^, Eduardo Salido^1^, Noel Mesa-Torres^2^, Angel L. Pey^2,^, and Mario Cano-Muñoz^2,3,*^.

^1^ Center for Rare Diseases (CIBERER), Hospital Universitario de Canarias, Universidad de la Laguna, 38320, Tenerife, Spain.

^2^ Departamento de Química Física, Unidad de Excelencia en Química Aplicada a Biomedicina y Medioambiente e Instituto de Biotecnología, Universidad de Granada, Av. Fuentenueva s/n, 18071, Granada, Spain.

^3^ Department of Biotechnology and Environmental Protection, Estación Experimental del Zaidín, Consejo Superior de Investigaciones Científicas, Granada 18008, Spain

Table of Contents

Table S12

Fig. S13

Fig. S24

Fig. S35

Table S2 …………………………………………………………………………………6

**Table S1 PLP binding equilibrium and kinetics to different apo-AGT variants.**

|  | **K_a_ (M^-1^)(10^5^)** | **K_d_ (μM)** | **k_on_ (M^-1^·s^-1^)** | **k_off_ (10^4^)(s^-1^)** |
| --- | --- | --- | --- | --- |
| **WT** | 1.7±0.4 | 5.9±1.4 | 95±3 | 5.6±1.2 |
| **p.S81A** | 2.4±0.6 | 4.2±1.1 | 132±3 | 5.4±1.2 |
| **p.S81D** | 0.029±0.003 | 345±35 | 4.7±0.3 | 17±1 |
| **LM** | 4.5±1.2 | 2.2±0.6 | 68±1 | 1.5±0.4 |
| **LM-p.S81A** | 1.7±0.3 | 6.0±1.1 | 79±2 | 4.7±0.8 |
| **LM-p.S81D** | 0.038±0.007 | 263±30 | 6.1±0.4 | 18±2 |
| **LM-p.G170R** | 2.4±0.5 | 4.2±0.9 | 70±1 | 2.6±0.7 |
| **LM-p.G170R/p.S81A** | 2.2±0.3 | 4.7±0.6 | 83±1 | 3.9±0.4 |
| **LM-p.G170R/p.S81D** | 0.043±0.012 | 240±70 | 8.0±1.5 | 19±2 |
| **LM-p.I244T** | 3.0±1.9 | 3.3±2.1 | 67±3 | 2.2±1.5 |
| **LM-p.I244T/p.S81A** | 1.9±0.6 | 5.3±1.7 | 74±2 | 3.8±1.1 |
| **LM-p.I244T/p.S81D** | 0.024±0.007 | 421±119 | 5.7±1.4 | 24±4 |

**Fig. S1 Conformation and specific activity of individual AGT proteins.** A) Far-UV CD AGT proteins as purified. For each variant, spectra are the average from two different purifications. B) Hydrodynamic radius for AGT proteins as purified from DLS analyses. Data are the average ± s.d. from 4-6 independent measurements. C) Overall transaminase activity of AGT variants using L-Alanine (100 mM) and glyoxylate (10 mM) as substrates. Data are the average ± s.d from at least four experiments.


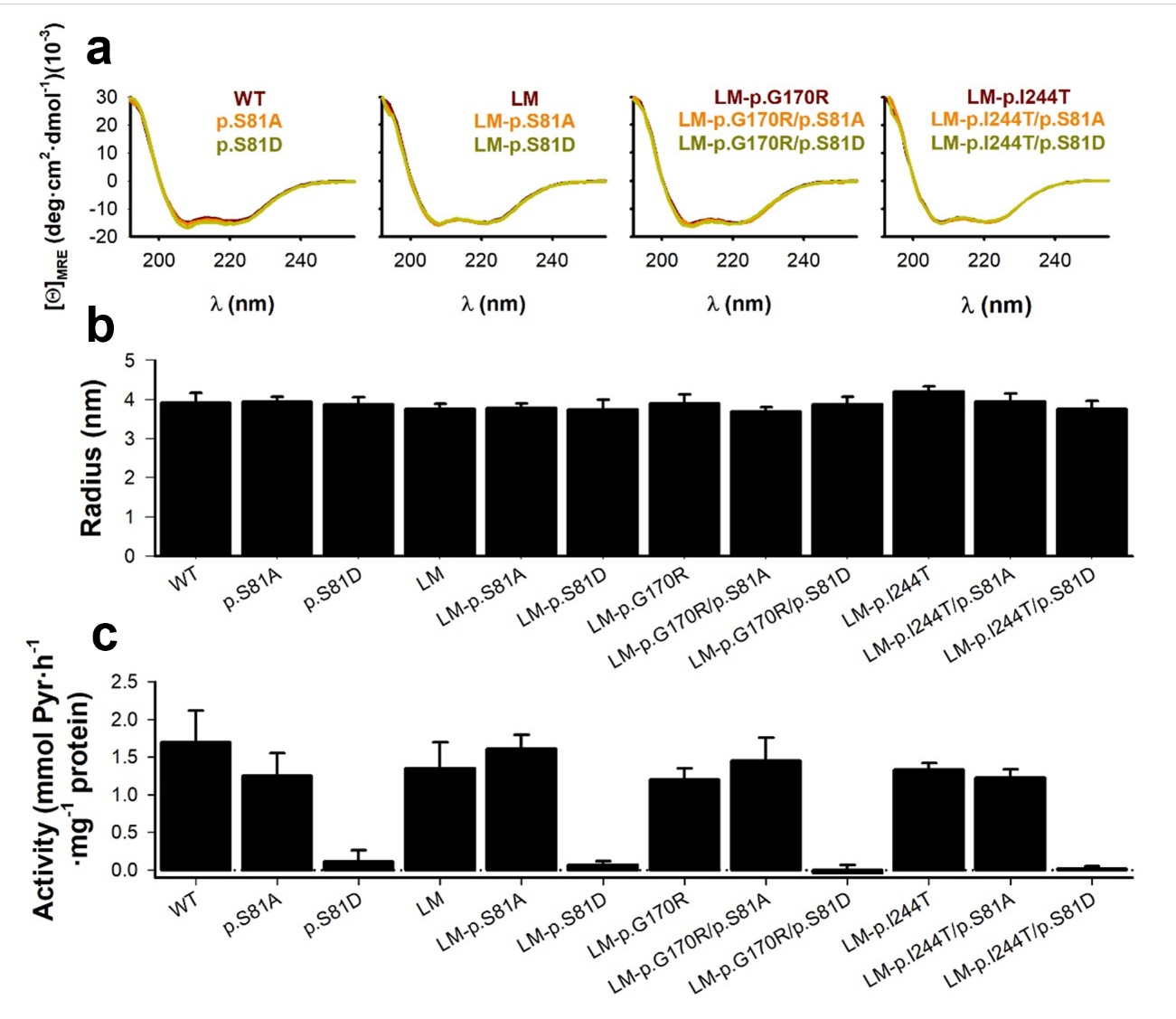


**Fig. S2 Thermal denaturation of holo- and apo-AGT individual variants.** A) Representative thermal scans. B) T_m_ values (mean±s.d. from four replicas).


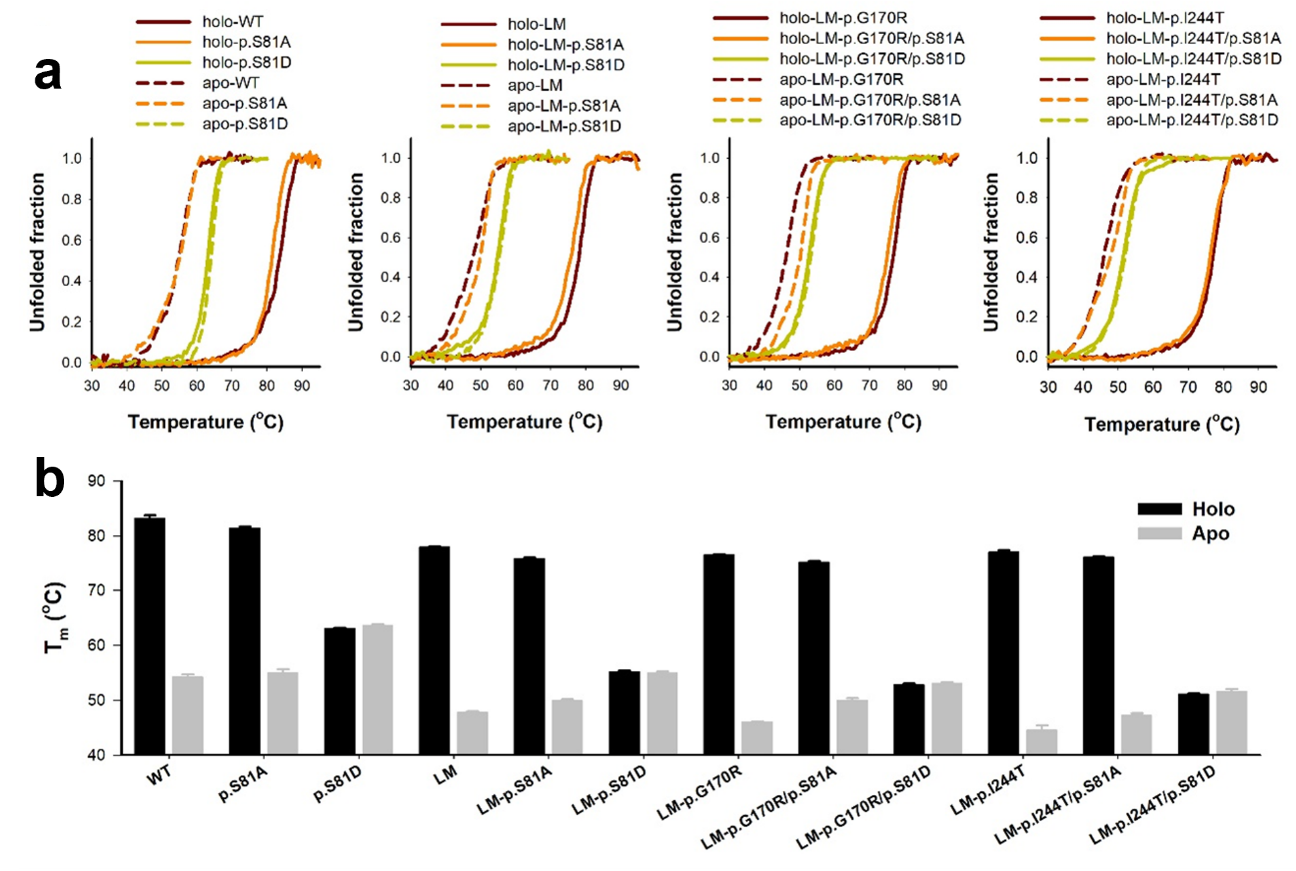


**Fig. S3 Spectroscopic characterization of PLP and PMP along the functional cycle of AGT variants.** A-B) Absorption (A) and CD (B) spectra of AGT proteins (15 μM) with and without incubation with alanine 200 mM. C) Absoption spectra of reaction mixtures containing AGT and alanine 200 mM (panel B) filtered using a 30 kDa concentration devices.


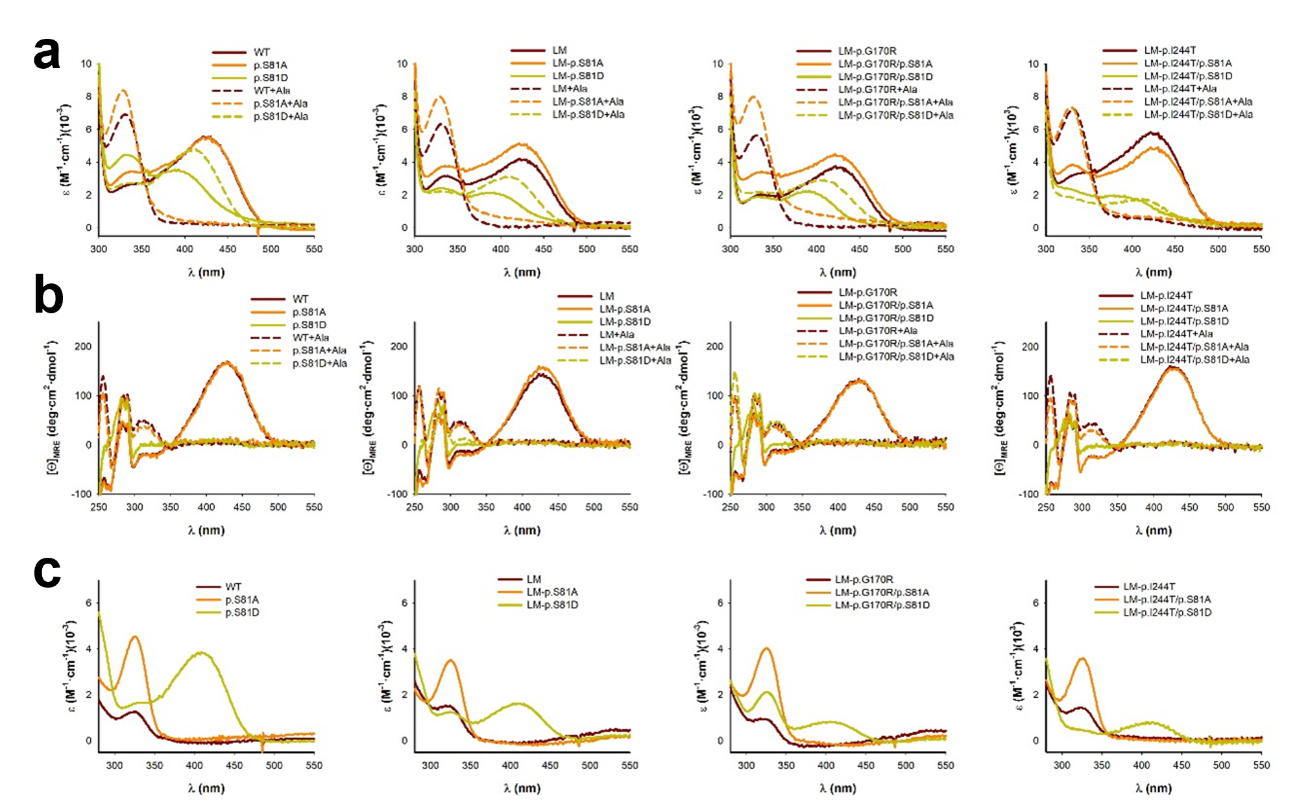


**Table S2 Quantitative colocalization análisis.** Analysis was done on Fiji (by ImageJ, https://imagej.net/software/fiji/), using Coloc 2 plugin for colocalization analysis. Photos were taken with Isis (by MetaSystems) software using 60x microscopic magnification.

|  | WT S81 AGT, PMP70 | WT S81 AGT, MIT | WT D81 AGT, PMP70 | WT D81 AGT, MIT | LM S81 AGT, PMP | LM S81 AGT, MIT | LM D81 AGT, PMP70 | LM D81 AGT, MIT |
| --- | --- | --- | --- | --- | --- | --- | --- | --- |
| Pearson's R (no threshold) | 0.590 | 0.230 | 0.547 | 0.303 | 0.580 | 0.294 | 0.440 | 0.242 |
| Pearson's R (above thr) | 0.410 | -0.163 | 0.290 | -0.150 | 0.320 | -0.131 | 0.090 | -0.216 |
| Manders' tM1 | 0.605 | 0.247 | 0.539 | 0.316 | 0.458 | 0.335 | 0.409 | 0.302 |
| Manders' tM2 | 0.633 | 0.350 | 0.557 | 0.389 | 0.517 | 0.371 | 0.433 | 0.287 |
| Spearman | 0.541 | 0.267 | 0.498 | 0.280 | 0.473 | 0.267 | 0.412 | 0.259 |
| Kendall | 0.383 | 0.192 | 0.356 | 0.200 | 0.346 | 0.193 | 0.297 | 0.185 |
| Li's ICQ | 0.243 | 0.147 | 0.226 | 0.139 | 0.244 | 0.130 | 0.199 | 0.153 |


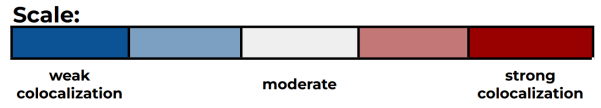


Legend for the statistics used:

| Pearson's R (no threshold) | R < 0.3 → weak colocalization | 0.3 ≤ R < 0.5 → moderate | 0.5 ≤ R < 0.7 → strong | R ≥ 0.7 → very strong |
| --- | --- | --- | --- | --- |
| Pearson's R (above threshold) | R < 0.3 → weak | 0.3 ≤ R < 0.5 → moderate | 0.5 ≤ R < 0.7 → strong | R ≥ 0.7 → very strong |
| Manders' tM1 | < 0.3 → very little overlap | 0.3-0.5 → moderate | 0.5-0.7 → substantial | > 0.7 → high |
| Manders' tM2 | < 0.3 → very little overlap | 0.3-0.5 → moderate | 0.5-0.7 → substantial | > 0.7 → high |
| Spearman's rank correlation value | < 0.3 → weak | 0.3-0.5 → moderate | > 0.5 → strong |  |
| Kendall's Tau-b rank correlation value | < 0.2 → weak | 0.2-0.35 → moderate | > 0.35 → strong |  |
| Li's ICQ | 0.0-0.1 → weak | 0.1-0.2 → moderate | 0.2-0.3 → substantial | > 0.3 → very strong |
